# BK Polyomavirus Micro-RNAs: Time Course and Clinical Relevance in Kidney Transplant Recipients

**DOI:** 10.3390/v13020351

**Published:** 2021-02-23

**Authors:** Baptiste Demey, Véronique Descamps, Claire Presne, Francois Helle, Catherine Francois, Gilles Duverlie, Sandrine Castelain, Etienne Brochot

**Affiliations:** 1 Laboratoire de Virologie, Centre Hospitalier Universitaire, F-80000 Amiens, France; descamps.veronique@chu-amiens.fr (V.D.); francois.helle@u-picardie.fr (F.H.); catherine.francois@u-picardie.fr (C.F.); gilles.duverlie@u-picardie.fr (G.D.); sandrine.castelain@u-picardie.fr (S.C.); 2 UR UPJV 4294, Agents Infectieux, Résistance et Chimiothérapie (AGIR), Centre Universitaire de Recherche en Santé, Université de Picardie Jules Verne, F-80000 Amiens, France; 3 Service de Néphrologie, Centre Hospitalier Universitaire, F-80000 Amiens, France; presne.claire@chu-amiens.fr

**Keywords:** polyomavirus BK, BKPyV, microRNA, kidney transplantation

## Abstract

Background: Kidney transplant recipients (KTRs) are exposed to a high risk of BK polyomavirus (BKPyV) replication, which in turn may lead to graft loss. Although the microRNAs (miRNAs) bkv-miR-B1-3p and bkv-miR-B1-5p are produced during the viral cycle, their putative value as markers of viral replication has yet to be established. In KTRs, the clinical relevance of the changes over time in BKPyV miRNA levels has not been determined. Methods: In a retrospective study, we analyzed 186 urine samples and 120 plasma samples collected from 67 KTRs during the first year post-transplantation. Using a reproducible, standardized, quantitative RT-PCR assay, we measured the levels of bkv-miR-B1-3p and bkv-miR-B1-5p (relative to the BKPyV DNA load). Results: Detection of the two miRNAs had low diagnostic value for identifying patients with DNAemia or for predicting DNAuria during follow-up. Seven of the 14 KTRs with a sustained BKPyV infection within the first year post-transplantation showed a progressive reduction in the DNA load and then a rapid disappearance of the miRNAs. DNA and miRNA loads were stable in the other seven KTRs. Conclusions: After the DNA-based diagnosis of BKPyV infection in KTRs, bkv-miR-B1-3p and bkv-miR-B1-5p levels in the urine might be valuable markers for viral replication monitoring and thus might help physicians to avoid an excessive reduction in the immunosuppressive regimen.

## 1. Introduction

BK polyomavirus (BKPyV) is a ubiquitous virus with a high seroprevalence in the general population [1]. After a frequently asymptomatic primary infection (caused by respiratory or oral-fecal transmission), BKPyV continues to replicate inconspicuously in urinary tract tissues. In the event of immunosuppression (as occurs in kidney transplant recipients (KTRs)), the risk of viral replication may increase [2]. Given the absence of preventive or curative antiviral treatment, a reduction in the immunosuppressive regimen decreases the risk of progression to polyomavirus-associated nephropathy (PyVAN) but accentuates the risk of graft rejection. A BKPyV DNA PCR assay is the standard method to diagnose and then monitor viral replication in KTRs [3]. Many studies have highlighted the benefits of BKPyV screening in the urine of KTRs to prevent PyVAN because viruria precedes BKPyV-DNAemia, especially for high-level viruria, when the urine BKPyV DNA load is higher than 7 log10 copies/mL [4]. Once BKPyV-DNAemia is proven, BKPyV DNA monitoring is less relevant in urine than in plasma. Moreover, the presence of BKPyV DNA is not always a sign of active viral replication, and DNA can sometimes be detected when defective virions are shed. Since KTRs are exposed to risk factors for kidney tissue damage [5], non-replicative BKPyV particles from dying cells might be released in the urine. Furthermore, slowly decreasing DNA concentrations can be detected in the urine for a long time after the diagnosis of BKPyV replication and the reduction in immunosuppression. In the absence of a clear DNA cut-off for a return to the initial immunosuppressive regimen, we need a specific, noninvasive marker of BKPyV replication so that full immunosuppression can be rapidly restored once the infection is under control.

Over the past few years, micro-RNAs (miRNAs, typically comprising 20–22 nucleotides) have emerged as promising diagnostic or prognostic markers for several diseases, including viral infections [6]. miRNAs can regulate protein expression by either binding directly to mRNA or by inhibiting the translational process through an RNA-induced silencing complex. BKPyV generates two mature miRNAs (bkv-miR-B1-3p and bkv-miR-B1-5p) during its replicative cycle, whose expression is driven by the Non-Coding Control Region (NCCR) [7]. The two miRNAs’ roles in the BKPyV life cycle are not well understood. Both miRNAs can induce the cleavage of BKPyV large tumor antigen (TAg) mRNA, which results in the autoregulation of viral replication. bkv-miR-B1-3p is also involved in escaping the immune response by targeting ULBP3 [8,9,10]. In vivo, BKPyV miRNAs have been found in various biological fluids, including urine, serum, plasma, and cerebrospinal fluid [11]. It has been reported that the BKPyV miRNA urine level is correlated with the BKPyV DNA load in KTRs. Moreover, BKPyV miRNAs may be useful non-invasive diagnostic markers for PyVAN [12]. However, data on BKPyV miRNAs in vivo are still scarce and are not easily comparable because the biological matrices (exosomes, cell pellets, or native urine) and the standardization methods differ from one study to another.

At present, the management of BKPyV infections in KTRs is based on periodic monitoring and early diagnosis. Then, the patient’s treatment is adapted in order to prevent irreversible tissue damage by PyVAN. DNA can be found in tissues and fluids even when viruses are inactive whereas miRNAs need an active viral cycle to be generated. Given that miRNAs might be more specific than DNA for viral replication, we hypothesized that measuring bkv-miR-B1-3p and bkv-miR-B1-5p levels in urine and/or plasma might help to track BKPyV infection changes over time in KTRs. Previous studies have measured BKPyV miRNA loads at single time points but not their changes over the course of the infection. Using standard literature methods, we developed an easy-to-use, reproducible RT-PCR assay to quantify BKPyV miRNA levels in urine and plasma.

In the present retrospective study, we measured BKPyV miRNAs in 186 urine samples obtained at various time points post-transplantation from 43 KTRs. The aim of the study was to determine thresholds of bkv-miR-B1-3p and bkv-miR-B1-5p amounts which could be used to predict, diagnose and monitor BKPyV replication in KTRs.

## 2. Materials and Methods

### 2.1. Study Design and Cohort

The study population comprised all the patients (*n* = 67) having undergone kidney transplantation between 5th July 2017, and 5th July 2018, at the Amiens University Medical Center (Amiens, France). The other main inclusion criteria were age 18 or older and regular follow-up at the medical center during the first year post-transplantation. Patients who experienced graft failure (transplant removal or requirement for dialysis) or who died during the follow-up period were excluded. Nineteen patients showed BKPyV DNA in urine during this period (hereafter referred to as “BKU cases”); of these, 17 had progressed to proven BKPyV-DNAemia (“BKP cases”). Twenty-four of the 48 patients with no BKPyV DNA detected during the first-year post-transplantation were randomly selected as controls. The present retrospective study was based on prospectively collected, de-identified biological samples and clinical information for the 43 selected KTRs (19 BKU cases and 24 controls). A total of 186 urine samples and 120 plasma samples were analyzed. The investigators who assayed the pairs of concomitantly collected urine and plasma samples for BKPyV miRNAs were blinded to the patients’ medical history and the BKPyV-DNAemia results. Regardless of case classification, the patients were categorized at each time point as U−P-− (undetectable BKPyV DNA in urine and plasma), U+P+ (DNA in both the urine and plasma), or U+P-− (DNA just in the urine). The samples were specimen overages obtained during standard care for KTRs at the Amiens University Medical Center. Demographic and clinical information was extracted from the medical center’s electronic medical records. In line with the French legislation on retrospective studies of routine clinical practice, approval by an institutional review board was not required. However, the study was registered with the French National Data Protection Commission (Commission nationale de l’informatique et des libertés (Paris, France)).

### 2.2. Nucleic Acid Extraction

Viral DNA was extracted from 200 μL of urine or plasma. 5 µL of a specific internal inhibition control (IC) was added to the lysis buffer and simultaneously purified with viral DNA by using specific protocol B in the NucliSENS easyMAG system (bioMérieux, Marcy-l’Etoile, France). The extracted DNA was eluted with 50 μL of elution buffer and frozen at −80 °C before use.

miRNAs were extracted from 400 µL of urine or plasma with miRNeasy Serum/Plasma Advanced Kit (Qiagen, Venlo, The Netherlands). The cel-miR-39 (miRBase accession number: MI0000010) was used as an IC and the standard for result normalization. cel-miR-39 (reference: MC10956) was obtained from Applied Biosystems (Foster City, CA, USA). 5.6 × 10^8^ copies of cel-miR-39 were added to each sample, as recommended by the manufacturer. The nucleic acids were extracted with a Qiacube device (Qiagen), according to the manufacturer’s protocol. The miRNAs were eluted with 30 µL of RNAse-free water, and the solution was frozen at −80 °C before use.

### 2.3. Real-Time PCR Assay for BKPyV DNA

BKPyV DNA was amplified and quantified with a RealStar BKV PCR Kit (Altona Diagnostics, Hamburg, Germany), as described previously [13].

### 2.4. miRNA RT-PCR Assay

All the kits, standards and thermocyclers used for the miRNA RT-PCR assays were supplied by Applied Biosystems.

Reverse transcription (RT): TaqMan^®^ MicroRNA Reverse Transcription Kit was used to reverse-transcribe BKPyV miRNAs and an IC, according to the manufacturer’s instructions. RT primers for miRNAs were provided with specific TaqMan MicroRNA Assays: 006801_mat for bkv-miR-B1-3p, 007796_mat for bkv-miR-B1-5p, and 000200 for cel-miR-39. For each miRNA and each sample, RT was performed with 5 µL of miRNA-enriched eluate and 10 µL of RT mix (prepared as recommended by the manufacturer). RT reactions were performed with a Veriti thermocycler, in accordance with the kit’s instructions. Complementary DNA (cDNA) specimens were frozen at −20 °C before use.

Real-time PCR: the reaction mix contained 1 µL of TaqMan MicroRNA Assay probes, 10 µL of TaqMan Universal PCR Master Mix, 7 µL of RNase-free water, and 2 µL of cDNA specimen. Real-time PCRs were performed in 96-well plates with the ABIPRISM 7900HT Sequence Detection System, as indicated in the TaqMan MicroRNA Assays manual. Signals were measured and analyzed using SDS 2.4.1 software (Applied Biosystems). Quantities in eluate were calculated from the cycle threshold (Ct) results, relative to standards diluted in the concentration range 3 × 10^1^ to 3 × 10^7^ molecules/microliter. The standards were the synthetic oligonucleotide sequences of bkv-miR-B1-3p, bkv-miR-B1-5p and cel-miR-39 (respectively UGCUUGAUCCAUGUCCAGAGUC, AUCUGAGACUUGGGAAGAGCAU and UCACCGGGUGUAAAUCAGCUUG; catalog numbers: MC14566, MC14356, and MC10956). The limit of detection for BKPyV miRNA in clinical samples was set to 1500 copies per reaction (i.e., 3.6 log10 copies per mL of the clinical sample), which corresponds to the minimal quantity of miRNA detected with reproducible signals after extraction and RT-PCR of 400 µL of a standard solution. The results were first normalized as the “quantity of cel-miR-39 in the miRNA-enriched eluate/standard quantity added before extraction (5.6 × 10^8^ copies)” ratio and then expressed as the number of copies (or log_10_ copies) per mL of urine or plasma.

### 2.5. Statistical Analyses

Statistical analyses were performed using GraphPad Prism version 8.0.0 for Windows (GraphPad Software, San Diego, CA, USA). Correlations between parameters were studied with Pearson’s test. The student’s t-test was carried out if the data were normally distributed; if not, a non-parametric Kruskal–Wallis test was performed. The area under the receiver operating characteristic (ROC) curve was calculated according to the Wilson/Brown method. The threshold for statistical significance was set to *p* < 0.05.

## 3. Results

### 3.1. Case-Control Analysis

The included patients were aged between 27 and 76 (median age: 55; mean age: 53.95), and the male:female ratio was 1.69:1. Analysis of the 186 urine specimens collected from the 43 KTRs revealed that BKPyV miRNAs were often not detected in the urine even when DNA was found: 29 of the 67 DNA-positive specimens tested negative for both miRNAs. In contrast, small quantities of BKPyV miRNAs were frequently observed in the immediate post-transplantation period (day 0 to 15), while the BKPyV DNA PCR assay was negative. Clinical data for controls, “BKU” cases (who showed BKPyV DNA in urine samples during the follow-up) and “BKP” cases (for whom BKPyV infection progressed to DNAemia) are summarized in Table 1. There was no statistically significant intergroup difference between cases and controls with regard to age, sex ratio, induction therapy (basiliximab vs. antithymocyte globulin) or maintenance treatment (tacrolimus vs. cyclosporine). Noteworthily, early detection of BKPyV miRNAs in urine between day 0 and day 15 post-transplantation was not associated with a higher risk of subsequent DNAuria (odds ratio (OR) [95% confidence interval (CI)] = 1.688 [0.507–6.376]) or DNAemia (OR [95% CI] = 1.143 [0.318–4.4351]) (not represented in Table 1).

### 3.2. Urine BKPyV miRNA Levels Are Less Accurate than DNA Levels as Marker of DNAemia

**Figure 1:**
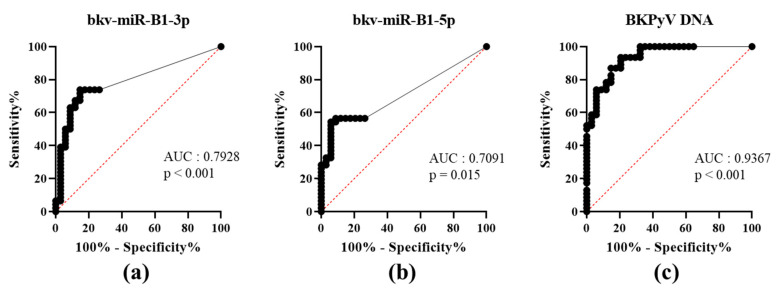
Receiver operating characteristic (ROC) curves for urine levels of bkv-miR-B1-3p (**a**), bkv-miR-B1-5p (**b**) and BKPyV DNA (**c**) in KTRs with (patients) vs. without (controls) BKPyV DNAemia at the time of sampling. AUC: area under the curve.

To date, PyVAN prevention is based on testing urine samples for high-level viruria [3]. This non-invasive screening option had a high negative predictive value. A viral load higher than 7 log_10_ copies/mL in the urine discriminated between patients with vs. without proven BKPyV-DNAemia [4]. To the best of our knowledge, the diagnostic value of using a BKPyV miRNA RT-PCR test to assay urine samples has not previously been evaluated. We analyzed bkv-miR-B1-3p, bkv-miR-B1-5p and BKPyV DNA concentrations in urine as a function of the corresponding plasma DNA status (patients: U+P+; or controls: U+P-) at the time of sampling and then plotted the associated ROC curves (Figure 1). The area under the curve (AUC) was high and statistically significant for both bkv-miR-B1-3p and bkv-miR-B1-5p (0.7928, *p* < 0.001; 0.7091, *p* = 0.015, respectively). bkv-miR-B1-3p and bkv-miR-B1-5p levels showed similar specificity, but bkv-miR-B1-3p was more sensitive as a marker of DNAemia. Compared to bkv-miR-B1-5p, bkv-miR-B1-3p average level was higher and might explain the better sensitivity performance to predict BKPyV-DNAemia. However, the BKPyV miRNA levels were less able than the urine DNA level (AUC = 0.9367, *p* < 0.001) to distinguish between patients with vs. without BKPyV-DNAemia (Table S1). The RT-PCR results showed that urine bkv-miR-B1-3p and bkv-miR-B1-5p concentrations were higher in KTRs with BKPyV-DNAemia (U+P+) than in KTRs without (U+P-) (Figure S1A). As expected, the urine levels of BKPyV DNA, bkv-miR-B1-3p and bkv-miR-B1-5p were correlated with the plasma BKPyV DNA level (Figure S1B). Hence, quantification of bkv-miR-B1-3p and bkv-miR-B1-5p in urine is less useful than BKPyV DNA loads for the diagnosis of BKPyV-DNAemia and the prevention of PyVAN.

### 3.3. BKPyV miRNAs: Changes over Time Following Kidney Transplantation

**Figure 2:**
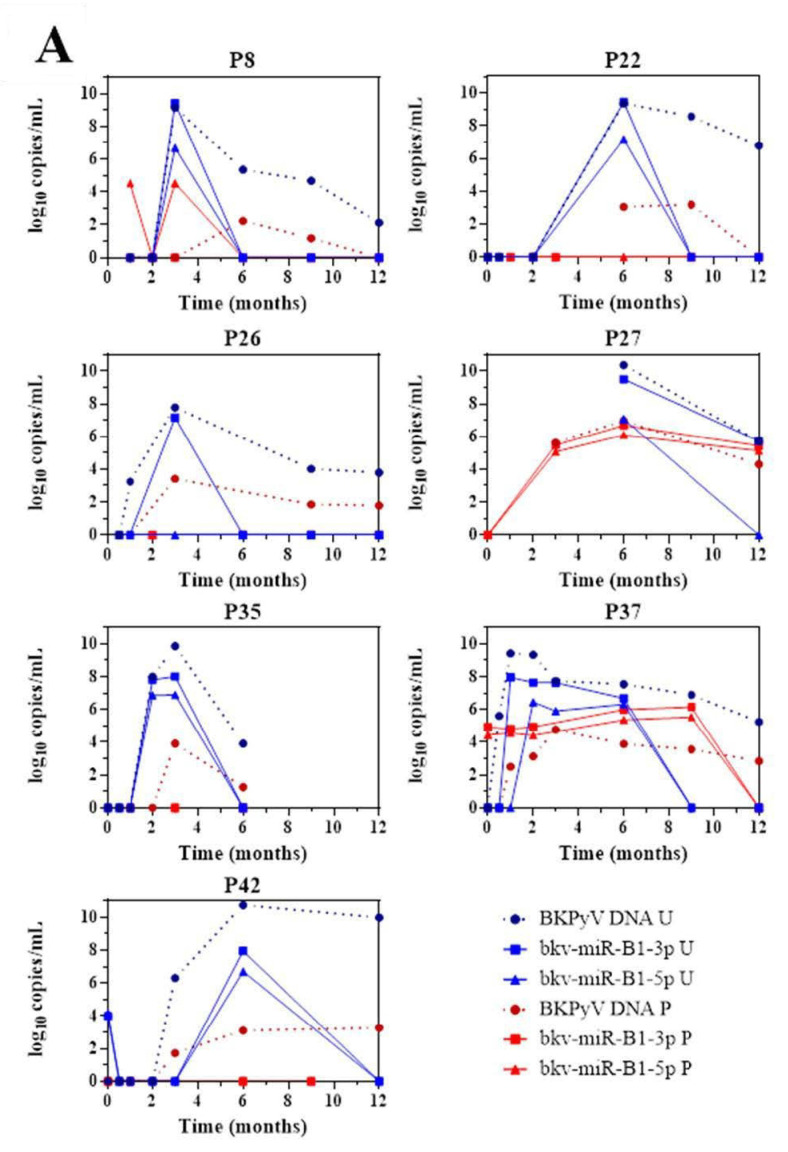

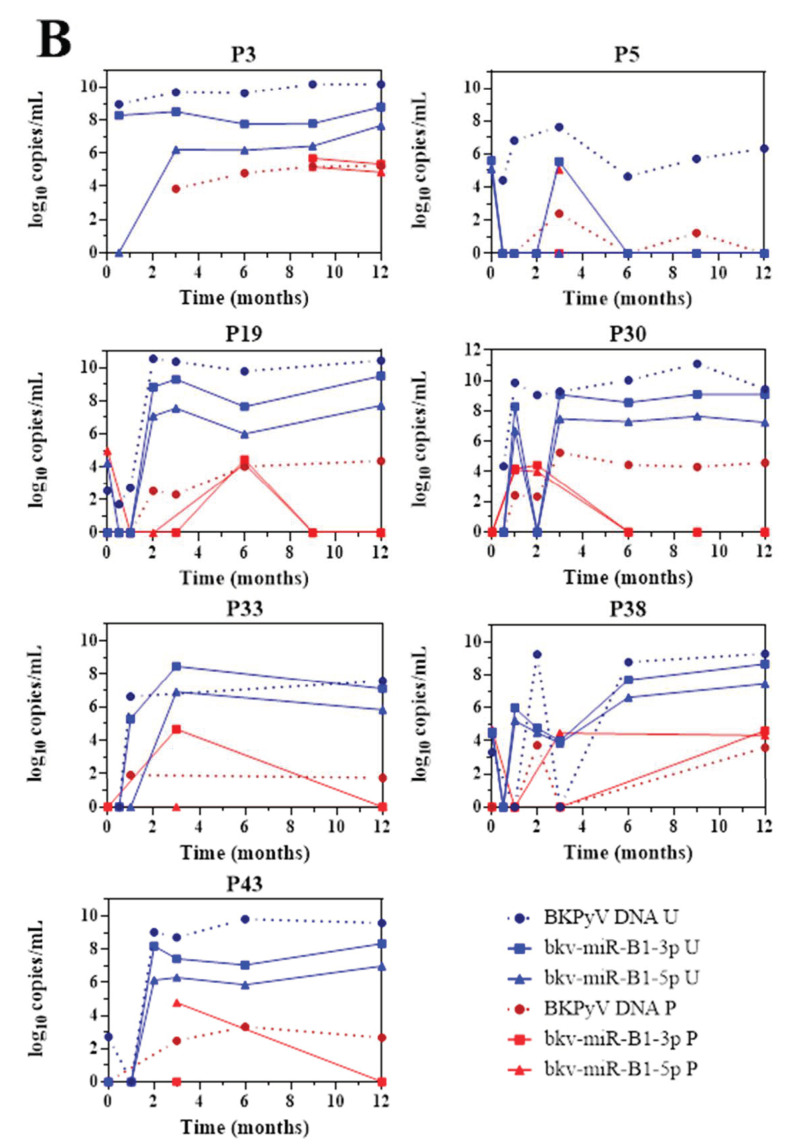
Changes over time of BKPyV markers (DNA, bkv-miR-B1-3p, bkv-miR-B1-5p) in urine (U) and plasma (P) among 14 patients who developed sustained BKPyV infection during the year after kidney transplantation. (**A**) Patients with a fall in the DNA load after immunosuppressant dose reduction. (**B**) Patients with a stable DNA load after immunosuppressant dose reduction.

The 43 KTRs of the study cohort had provided samples on a regular basis throughout the first 12 months post-transplantation. Fourteen of the 19 BKU cases were diagnosed with sustained BKPyV infection (two or more consecutive detections of BKPyV-DNA) during this period. Firstly, we quantified the patients’ urine and plasma levels of bkv-miR-B1-3p, bkv-miR-B1-5p, and BKPyV DNA at different time points (Figure 2). Overall, the changes over time were similar for miRNAs and DNA, and the two concentrations peaked at the same time. Furthermore, bkv-miR-B1-3p was detected more readily than bkv-miR-B1-5p in urine: the bkv-miR-B1-3p concentration was always higher than the bkv-miR-B1-5p concentration, and bkv-miR-B1-3p was sometimes present when bkv-miR-B1-5p was not (at month 3 for patient 26, for example). In contrast, more plasma samples were positive for bkv-miR-B1-5p (21 out of 57) than for bkv-miR-B1-3p (15 out of 57). Interestingly, the urine levels of BKPyV miRNAs fell rapidly (sometimes to zero) in the 7 patients who presented a slight decrease in the DNA load after immunosuppression reduction (P8, P22, P26, P27, P35, P37 and P42) (Figure 2A). In these cases, BKPyV DNA cleared slowly and was detected long after the miRNAs had disappeared. For example, P22 clearly showed a rapid fall to zero of urine miRNA levels 3 months after BKPyV infection diagnosis and immunosuppression reduction. Meanwhile, gold standard plasma DNA qPCR remained positive 6 months and slowly decreased. The profiles of the curves for P8, P27 and P42 differed marginally from the others. BKPyV-DNAemia seemed to appear at month 6 post-transplantation when BKPyV-DNAuria regressed, and miRNAs disappeared for P8. This might be explained by a missing plasma sample before the 6^th^ month, when plasmatic BKPyV-DNA level was higher than 1 log10 copies/mL. BKPyV-DNAuria and BKPyV-DNAemia barely declined through time for P27 and P42 but BKPyV-DNA load was negative in plasma at month 14 (data not shown). As for P8, we hypothesized that a missing higher DNA load peaked in plasma within these periods but could not be observed. For 7 other BKU cases, DNA and miRNA loads in urine remained stable and kinetics were parallel (Figure 2B). Singular kinetics were found for P26: a low bkv-miR-B1-3p level was detected at the same time as the highest DNA loads in urine and plasma. After this time, BKPyV miRNAs were undetectable but DNA persisted in urine and plasma until month 15 (time point not shown on Figure 2B), which explained P26 classification as one of the 7 stable patients. Low miRNA level, below the limit of detection, was a presumed rationale. For the other 5 BKU patients, BKPyV DNAuria was diagnosed during the 12^th^-month post-transplantation and could not be included in the primary analysis of sustained BKPyV infections during the 1-year follow-up period. We were therefore able to obtain urine specimens collected 1 to 4 months after the first detection of BKPyV. Four of the 5 patients presented a low DNA load and no bkv-miR-B1-3p (Figure S3). These extended experiments thus confirmed our primary observations. Ultimately, we found that the time course of miRNA levels differed from that of DNA in most KTRs with sustained BKPyV replication. Given that miRNAs are more specific markers of active replication than viral DNA, we suggest that miRNA monitoring might be more useful than DNA to track the BKPyV replication standstills after immunosuppressant dose reduction in KTRs.

## 4. Discussion

The roles of bkv-miR-B1-3p and bkv-miR-B1-5p in the physiopathology of BKPyV infection are not well understood, and so the interpretation of their presence and levels in vivo is challenging. Previous research highlighted the presence of BKPyV miRNAs in specific matrices (exosomes, cell pellets from urine, or plasma) but focused on precise stages of the disease (BKPyV-DNAemia or PyVAN) [8,14,15,16]. Here, we developed a standardized method to easily measure BKPyV miRNA concentrations in non-preprocessed biological fluids, in order to evaluate the analytes’ diagnostic and prognostic value. The analysis of whole urine and whole plasma was preferred to the analysis of enriched exosomal fractions to avoid bias. In fact, the literature does not contain any data about the percentage of extracellular BKPyV miRNAs included in exosomes. Surprisingly, we were not confronted with the false—positive RT-PCRs (Ct > 35 for control reactions with no template) previously described for the same assay kit [11,12]. We retrospectively analyzed 186 urine samples and 120 plasma samples collected from 43 KTRs over the course of the first 12 months post-transplantation. This constituted the largest cohort of specimens and patients assessed for BKPyV miRNAs. As mentioned in the Introduction, the objective of the present study was to evaluate changes over time in bkv-miR-B1-3p and bkv-miR-B1-5p RT-PCR results during the first 12 months post-transplantation and thus throughout the different stages of BKPyV replication. Like Huang et al., we found that the urine miRNA concentrations were higher in patients with proven BKPyV-DNAemia than in patients without [12]. We also confirmed that the bkv-miR-B1-3p and bkv-miR-B1-5p levels in urine correlated with BKPyV DNA levels in urine and plasma. While Huang et al. did not detect BKPyV miRNAs in plasma; we observed bkv-miR-B1-3p and bkv-miR-B1-5p in respectively 15 and 21 of the 57 plasma samples from the 14 patients with sustained BKPyV replication. We found that plasma BKPyV DNA levels correlated with plasma bkv-miR-B1-3p levels but not with plasma bkv-miR-B1-5p levels (Figure S2). This observation mainly results from three positive bkv-miR-B1-5p RT-PCR assays in plasma samples from patients without DNAemia. We were not able to determine whether these were true or false positives. On one hand, a false-positive RT-PCR test might be caused by the presence of interferents in the plasma. On the other hand, a negative miRNA RT-PCR test in a plasma sample from a viremic patient might be due to insufficient sensitivity or to declining viral replication. As we suggest that BKPyV miRNA and DNA levels provide different information about the infection, our experiments cannot solve this problem for the moment.

We noticed that with regard to urine samples, 46% of the KTRs were positive for BKPyV miRNAs but negative for viral DNA during the first two weeks post-transplantation. This period follows induction therapy and features the highest doses of immunosuppressive maintenance therapy—providing a “breeding ground” for viruses like BKPyV. We hypothesized that BKPyV miRNAs might be valuable markers of viral replication and thus might enable early diagnosis. However, the detection of bkv-miR-B1-3p and bkv-miR-B1-5p in urine before day 15 post-transplantation was not associated with a higher risk of subsequent BKPyV DNAuria.

We next compared the urine BKPyV miRNA and DNA levels with the plasma DNA levels. This enabled us to compare urine miRNA screening with urine DNA screening—one of the most common means used to prevent PyVAN in KTRs. Urine miRNA screening was less efficient than urine DNA screening to diagnose DNAemia, with sensitivities of 73.3%, 55.6% and 100% for bkv-miR-B1-3p, bkv-miR-B1-5p, and BKPyV DNA, respectively. However, the specificity was above 80% for both bkv-miR-B1-3p and bkv-miR-B1-5p—even at low miRNA concentrations. Better BKPyV miRNA purification and RT-PCR is required before this method can be considered as a replacement for BKPyV DNA screening in urine.

As already mentioned in the literature, BKPyV miRNA assays might be of value for monitoring BKPyV infections [12]. The presence of miRNAs reflects gene expression, whereas the presence of DNA might correspond to the clearance of defective virions or the passive shedding of latently infected cells into the urine. TAg or VP1 mRNAs were also candidates for replication monitoring but they are less resistant in the extracellular environment. miRNA quantification is more practical because it requires fewer preprocessing steps and can be performed on the sample used for DNA analysis [17,18]. At present, the only treatment for BKPyV replication in a KTR is an immunosuppressant dose reduction, which increases the risk of graft rejection. Physicians lack a specific marker of a controlled infection and thus the opportunity to restore a full immunosuppressive regimen. Our results highlighted that of the 14 patients who showed sustained BKPyV replication before the 12th-month post-transplantation, seven controlled the replication: the BKPyV DNA level fell progressively after the dose reduction. All seven patients cleared BKPyV miRNAs quickly, which might indicate that viral replication had already stopped. Four of the other five BKU cases showed similar changes over time but the observation period was shorter and the changes (based on just two measurement time points) were not as well-defined. Martelli et al. reported similar variations over-time for the BKPyV DNA load and exosomal bkv-miR-B1-5p levels in urine samples from multiple sclerosis patients, depending on NCCR rearrangements [16]. Even for the seven patients with stable DNA loads through time, none of the included KTRs developed a proven PyVAN in the present study. The missing samples and restricting information about treatment adjustment limited our analyses of changes over time for BKPyV markers. Further prospective studies focusing on peaks and troughs in BKPyV markers, and NCCR rearrangements through time in a larger cohort of KTRs might provide more robust evidence. In the future, bkv-miR-B1-3p and bkv-miR-B1-5p might constitute predictive markers of a slowing or standstill in viral replication, in addition to gold-standard BKPyV DNA qPCR assay. Therefore, BKPyV miRNAs might be interesting indicators to evaluate the infection prognosis. If so, this would help physicians to limit the duration of immunosuppressant dose reduction, even when the BKPyV DNA titer is still high.

An important issue of BKPyV miRNA screening is the identical structure between bkv-miR-B1-3p and jcv-miR-J1-3p, a mature miRNA produced by JC Polyomavirus (JCPyV). Given that bkv-miR-B1-3p was more often detected than bkv-miR-B1-5p in urine of KTRs, we checked in the samples whether JCPyV DNA was also detectable. We only found JC polyomavirus DNA in two samples (both from the same patient); it was associated with urine bkv-miR-B1-3p concentrations of 7.8 and 6.3 log_10_ copies/mL while bkv-miR-B1-5p and BKPyV DNA were undetectable. Thus, bkv-miR-B1-3p should not be screened if not associated with bkv-miR-B1-5p RT-PCR assay. Moreover, simultaneous shedding of JCPyV and BKPyV DNA in urine is rare [19]. After the initial diagnosis of BKPyV infection based on viral DNA detection, a combined bkv-miR-B1-3p and bkv-miR-B1-5p monitoring would not be affected by false-positive results and interpretation issues caused by a presumed simultaneous JCPyV infection. Nevertheless, the discordance between bkv-miR-B1-3p and bkv-miR-B1-5p levels in a sample could be caused by the inconsistent performance of the two RT-PCR assays, as for P19 and P30. An enhancement of sensitivity performance of bkv-miR-B1-5p RT-PCR assay is needed to make it a specific and valuable single marker of active BKPyV replication. In fact, the low sensitivity of bkv-miR-B1-5p RT-PCR might induce false negative results which could be misinterpreted as a shutdown BKPyV replication. There is a probability that concerned the results for P27 at month 12.

Recent research has clarified the diagnostic utility of miRNAs for BKPyV replication in KTRs. The value for PyVAN diagnosis has already been described. Here, we studied the value of BKPyV miRNAs for early diagnosis and specific monitoring of active viral replication. Our results revealed urine bkv-miR-B1-3p and bkv-miR-B1-5p levels are potential markers of viral replication in KTRs, but currently have low diagnostic value. However, these markers might enable a more rapid re-establishment of the full immunosuppressive regimen after BKPyV control and might thus prevent graft rejection. Given that BKPyV can be cultured in vitro for several weeks, long-term culture experiments might confirm our present in vivo observations in a large cohort of KTRs; [20]. The quantification of miRNAs and DNA in cells and conditioned media might help to characterize the changes over time in these markers and thus confirm the phenomena observed in the present clinical study.

## Figures and Tables

**Table 1:** Clinical data for kidney transplant recipients (KTRs).

		Cases		*p* Value	
Patients	Controls	BKU	BKP	Controls vs. BKU	Controls vs. BKP
*n* (total = 43)	24	19	17		
Age (mean ± SEM)	52.29 ± 2.66	56.05 ± 3.30	58.00 ± 3.28	0.375	0.182
Male:female sex ratio	1.4	2.17	2.4	0.542	0.52
Induction therapy					
Basiliximab (vs. ATG)	16 (vs. 8)	9 (vs. 10)	8 (vs. 9)	0.23	0.335
Maintenance therapy					
Tacrolimus (vs. Cyclosporine)	21 (vs. 3)	19 (vs. 0)	17 (vs. 0)	0.242	0.254

Controls: patients without a diagnosis of BKPyV replication during the follow-up. BKU: patients with BKPyV DNAuria during the follow-up. BKP: patients with proven BKPyV-DNAemia during the follow-up (all of whom were included in the BKU group). SEM: standard error of the mean.

## Data Availability

The data presented in this study are available on request from the corresponding authors. The data are not publicly available due to ethical restrictions.

## Supplementary Materials

The following are available online at https://www.mdpi.com/1999-4915/13/2/351/s1, Figure S1: Urine levels of bkv-miR-B1-3p. bkv-miR-B1-5p and BKPyV DNA when BKPyV DNA was also found in plasma (U+/P+) at the same time or not (U+/P-), Figure S2: Correlation between the plasma BKPyV DNA load and the plasma concentration of bkv-miR-B1-3p or bkv-miR-B1-5p, Figure S3: Changes over time in levels of BKPyV markers in urine (U; DNA, bkv-miR-B1-3p and bkv-miR-B1-5p) and plasma (P; DNA) for 4 KTRs who developed BKPyV replication 12 months post-transplantation Table S1: Sensitivity and specificity analyses for urine levels of bkv-miR-B1-3p, bkv-miR-B1-5p and BKPyV DNA in KTRs with (patients) or without (controls) BKPyV DNAemia at the time of sampling.
