# Supplementary material for: BK Polyomavirus Micro-RNAs: Time Course and Clinical Relevance in Kidney Transplant Recipients
Source: Viruses. 2021 Feb 23;13(2):351. doi: 10.3390/v13020351 (PMC7926448; doi:10.3390/v13020351)
Supplement: Supplementary file 1 [file viruses-13-00351-s001.pdf]

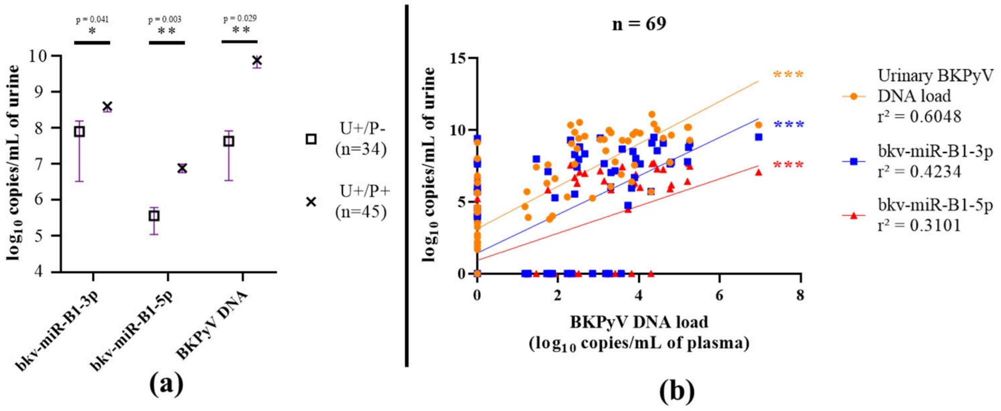

Figure S1. (a): Urine levels of bkv-miR-B1-3p. bkv-miR-B1-5p and BKPyV DNA when BKPyV DNA was also found in plasma (U+/P+) at the same time or not (U+/P-). (b): Correlation between the plasma BKPyV DNA load and the urine concentration of three BKPyV markers (DNA, bkv-miR-B1-3p and bkv-miR-B1-5p). *** indicates statistical significance with $p < 0.001$.

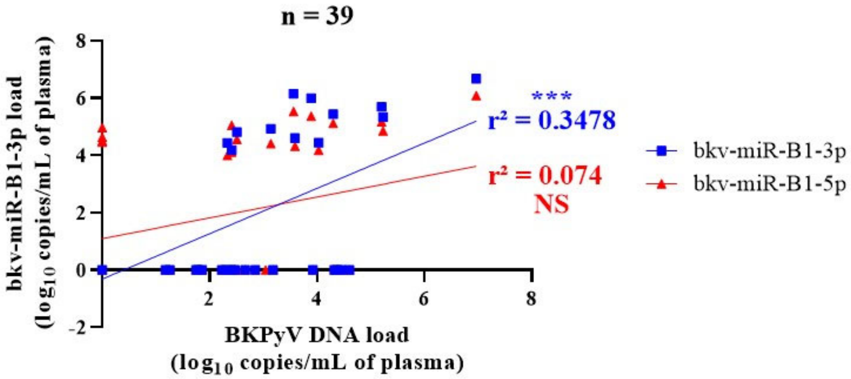

Figure S2. Correlation between the plasma BKPyV DNA load and the plasma concentration of bkv-miR-B1-3p or bkv-miR-B1-5p. *** indicates statistical significance with $p < 0.001$. NS: not significant.

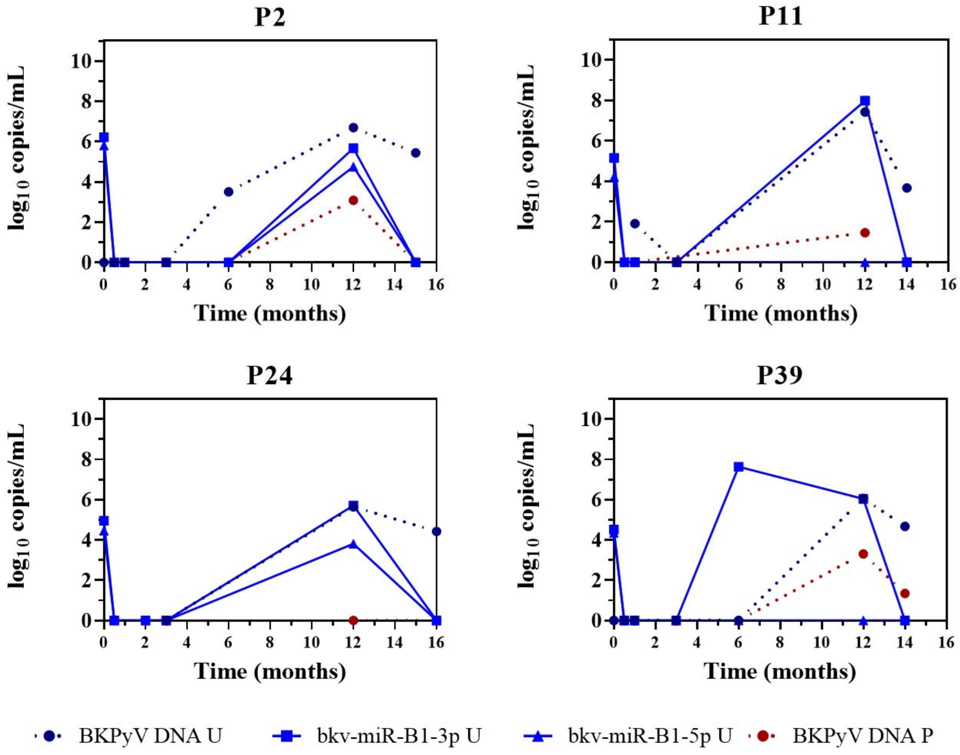

Figure S3. Changes over time in levels of BKPyV markers in urine (U; DNA, bkv-miR-B1-3p and bkv-miR-B1-5p) and plasma (P; DNA) for 4 KTRs who developed BKPyV replication 12 months post-transplantation.

		BKPyV marker		
		bkv-miR-B1-3p	***bkv-miR-B1-5p***	***BKPyV DNA***
**Maximum sensitivity**	**Sensitivity**	73,3 %	55,56 %	100 %
	**Specificity**	85,3 %	91,2 %	4,46
	**Urine level (log copies/mL)**	4,65	4,46	3,65
**Maximum specificity**	**Sensitivity**	6,67 %	28,9 %	51,1 %
	**Specificity**	100 %	100 %	100 %
	**Urine level (log copies/mL)**	9,42	6,88	9,18
**Maximum likelihood ratio**	**Sensitivity**	62,2 %	53,3 %	73,3 %
	**Specificity**	91,2 %	94,1 %	94,1 %
	**Urine level (log copies/mL)**	6,02	5,53	6,86

Table S1. Sensitivity and specificity analyses for urine levels of bkv-miR-B1-3p, bkv-miR-B1-5p and BKPyV DNA in KTRs with (patients) or without (controls) BKPyV DNAemia at the time of sampling.
